# Biophysical models of dispersal contribute to seascape genetic analyses

**DOI:** 10.1098/rstb.2021.0024

**Published:** 2022-03-14

**Authors:** Marlene Jahnke, Per R. Jonsson

**Affiliations:** Department of Marine Sciences, Tjärnö Marine Laboratory, University of Gothenburg, Strömstad 45296, Sweden

**Keywords:** seascape genetics, seascape genomics, biophysical modelling, population genetics, dispersal

## Abstract

Dispersal is generally difficult to directly observe. Instead, dispersal is often inferred from genetic markers and biophysical modelling where a correspondence indicates that dispersal routes and barriers explain a significant part of population genetic differentiation. Biophysical models are used for wind-driven dispersal in terrestrial environments and for propagules drifting with ocean currents in the sea. In the ocean, such seascape genetic or seascape genomic studies provide promising tools in applied sciences, as actions within management and conservation rely on an understanding of population structure, genetic diversity and presence of local adaptations, all dependent on dispersal within the metapopulation. Here, we surveyed 87 studies that combine population genetics and biophysical models of dispersal. Our aim was to understand if biophysical dispersal models can generally explain genetic differentiation. Our analysis shows that genetic differentiation and lack of genetic differentiation can often be explained by dispersal, but the realism of the biophysical model, as well as local geomorphology and species biology also play a role. The review supports the use of a combination of both methods, and we discuss our findings in terms of recommendations for future studies and pinpoint areas where further development is necessary, particularly on how to compare both approaches.

This article is part of the theme issue ‘Species’ ranges in the face of changing environments (part I)’.

## 1. Background

**Figure 1.**
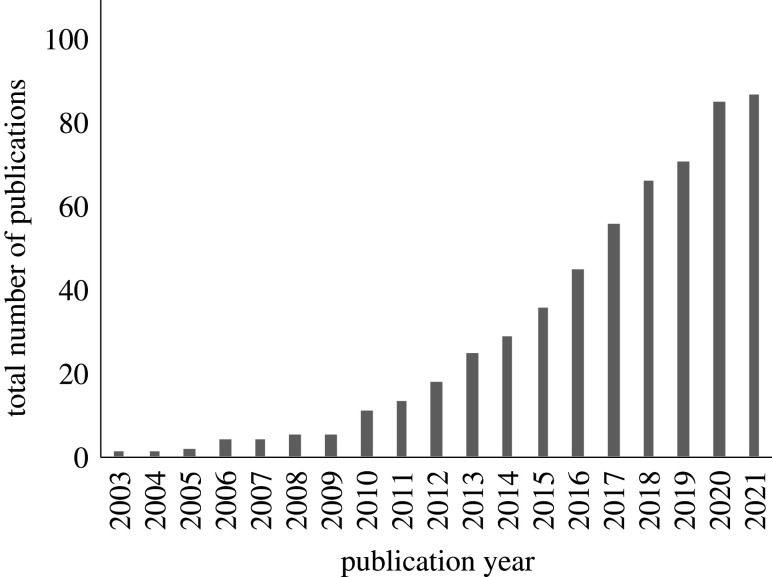
Cumulative appearance of surveyed publications that combine genetics and biophysical modelling in a seascape context. See the electronic supplementary material for the full reference list.

Few species exist as coherent and panmictic populations. Instead, species are often patchily distributed reflecting underlying habitats, barriers to dispersal and local extinctions. Species distributions are also shifting and being fragmented, caused by multiple human pressures leading to habitat loss [1]. Local populations may interact as a metapopulation through dispersal leading to the exchange of gametes, seeds or individuals [2]. Metapopulation dynamics have potentially large and varied implications for demography and population persistence, and also for genetic differentiation and evolution of local adaptation with potential for incipient speciation [3]. Dispersal and metapopulation connectivity are thus key for the understanding of ecology and evolution of most species. Dispersal is also involved in range expansion and range shifts, e.g. caused by ongoing climate change [4]. Understanding species distributions and population expansions in the context of environmental heterogeneity and a geographical context requires a landscape approach [5]. Landscape genetics adds information on genetic structure and genetic connectivity to understand how environmental parameters influence the extent of genetic variation within and among populations [6]. When studies are carried out in the sea, these studies are defined as seascape genetic studies [7]. Seascape genetics is still a young discipline, with the first use of the term in a publication in 2006 [8]. The first paper that used a combination of genetics and oceanography was already published before the term was coined, in 2003 [9], but the majority of studies have only been published in the last 10 years (figure 1). With the advent of genomics, the discipline of seascape genomics has also developed, which additionally allows studies of local adaptation through field-based investigations [5].

Seascape genetics and seascape genomics meet at the interface between basic and applied sciences. The knowledge that comes out of such studies can provide important information for the management and protection of resources, ecosystems and species. For instance, Jahnke *et al*. [10] used a seascape genetic approach for seagrass in a fjord-scale study in Sweden to define management units, provided a list of particularly valuable meadows (based on genetic and biophysical connectivity) that require protection and even suggested ideal restoration candidate sites to improve dispersal probabilities within the metapopulation [10]. Similarly, Matz *et al*. [11] used seascape genomics, biophysical modelling and evolutionary simulations to predict the future adaptation of a coral on the Great Barrier Reef in Australia [11]. This study was able to show that dispersal plays a crucial role in future persistence and adaptation, suggesting management interventions such as assisted gene flow [12] by moving adult reproductively active colonies or by out-planting laboratory-reared offspring produced by crossing corals from different populations [11]. Seascape genomics also has important value in fisheries management. Silva *et al*. [13] applied a seascape genomics approach to provide information on source-sink dynamics of rock lobster on large spatial scales, with important implications for fisheries management of the stock and predictions on how recruitment could be altered under future environmental conditions [13]. A crucial point here is that none of these studies would have been as meaningful without the combined approach of genetics and biophysical modelling, as both approaches are able to complement as well as validate each other. The genetic assessments may integrate over many gene-flow mechanisms and capture regional population history through deep time, or assess single dispersal events or the time depth of one to four generations (i.e. detecting ancestry down to a quarter of the genome) with assignment tests [14] and relatedness assessments [15,16]. Crucially, diversity metrics and population differentiation, as well as inferences about demography, can only be determined with genetic or genomic data [17]. Biophysical modelling of virtual particles in sea current models instead provides insights about single or multiple generations of dispersal and how the metapopulation is shaped by circulation patterns and dispersal barriers. Biophysical models can also offer high resolution in space and time, but the main shortcoming is that they cannot deliver information about demographic history or adaptive processes (including selective processes such as larval exhaustion and genotype by environment mismatches) of populations or species [17]. The added value of using both genetics/genomics and biophysical approaches strengthens conservation planning and eventual monitoring of a particular management plan, because once confidence is gained in the biophysical model, it is possible to rerun it with different data and under different scenarios to reflect adaptive management [18]. Together the two methods can provide insights on differences between demographic, functional and neutral genetic connectivity, and have the power to provide important and reliable information on applications to marine reserve design, fisheries science and strategies to assess resilience to climate change and other anthropogenic impacts [8]. Clearly, several publications of the past decade testify to the value of combining population genetics and biophysical modelling in a seascape framework. In this paper, we aim to review the available literature to explore if biophysical models are generally successful in explaining patterns of population genetics in the seascape, and if this depends on certain aspects of the seascape, biology, data and models.

## 2. Survey of seascape genetic studies

**Figure 2.**
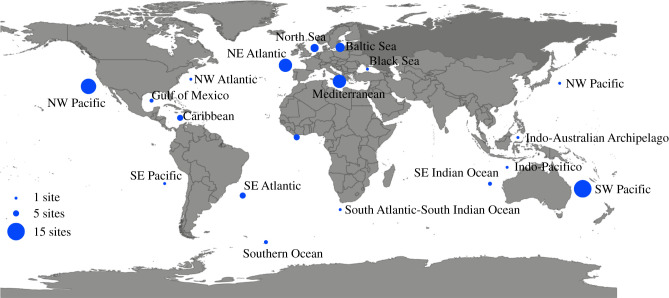
Major oceans and seas, with circle size proportional to number of studies in each body of water. Additionally, two global studies were included in this survey. (Online version in colour.)

We surveyed the seascape genetic and seascape genomic literature and searched for publications in February 2021 with the following keywords: ‘biophysical modelling, population genetics’, ‘dispersal, population genetics, marine’ and ‘seascape genomics’ on Web of Science and Google Scholar and retained all papers that used biophysical modelling as well as population genetic/genomic approaches. Some additional papers were added after broad reading of the literature by the authors. This resulted in 87 papers that were included in our database of publications that use biophysical and genetic approaches together. We note that those 87 papers that use both population genetic and biophysical approaches are a subset of the growing field of seascape genetics/genomics that also includes investigations into adaptation to heterogeneous local conditions and habitat availability [19]. As several publications assessed multiple species or several marker types, 103 different assessments were made. A full reference list can be found in the electronic supplementary material, SA1 and SA2. The 87 papers assessed seas and oceans to an equal degree. The South West Pacific (16 papers), the North East Pacific (14 papers) and the North East Atlantic and Mediterranean (12 papers) were the most studied areas, but large parts of the world's oceans and seas are very underrepresented with few studies from South America, Africa, Asia and the polar regions (figure 2).

**Figure 3.**
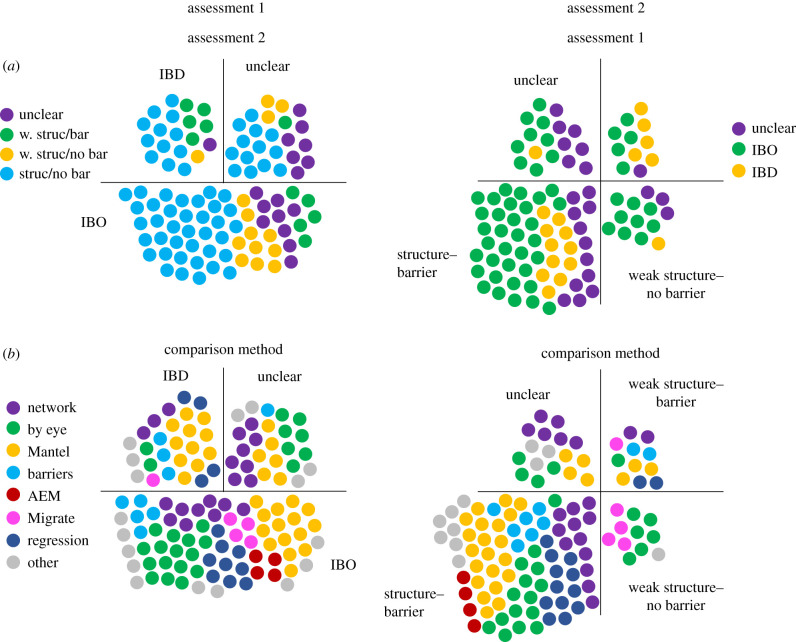
Plots of the classification scheme in assessment 1 (table 1) and assessment 2 (table 2) of the 103 cases. Shown in (*a*) is how the cases classified in assessment 1 are classified in assessment 2 and vice versa (w. struc/bar, ‘weak structure–barrier’; w. struc/no bar, ‘weak structure–no barrier’; struc/bar ‘structure–barrier’). Shown in (*b*) are the methods used for the studies concluding fit according to our assessments 1 and 2. (Online version in colour.)

We then surveyed the 103 assessments further to understand under what conditions there is a good fit between genetics and biophysical modelling. This fit was assessed in a qualitative rather than a quantitative way, as (i) there is a wide heterogeneity of the methods used and parameters included in the different studies, so only a handful of papers each would be comparable enough for a formal meta-analysis; and (ii) it is challenging to define a null hypothesis. In some publications, isolation-by-distance (IBD, which correlates genetic differentiation to the geographical distance among sites) is considered a null-model to which the correlation coefficient for isolation-by-oceanography (IBO, which correlates genetic differentiation to dispersal probability) is compared. These authors would argue that if IBO results in a significantly higher correlation this indicates that the biophysical model explains some added portion of the total variation in the genetic signal. We agree, but also argue that if IBO gives a similar correlation (but not higher) the biophysical model also offers a *mechanistic* explanation to the observed IBD, as other processes than dispersal, particularly colonization and local adaptation, can lead to genetic differentiation along a spatial gradient, i.e. IBD [20,21]. In addition, we also argue that even if there is no significant IBD and also no correlation for IBO, this can be seen as a good fit between high gene flow and extensive dispersal, and again the biophysical model has offered some insight about the observed pattern. To be able to nevertheless compare the heterogeneous publications and consider any fit between genetic structure and modelled dispersal, we classified the papers in two ways for possible fit: assessment 1, according to whether biophysical dispersal probability explains genetic structure better than IBD (table 1), and assessment 2, a matching classification, assessing whether both methods agreed in finding—or not finding—structure (table 2), i.e. describing in a qualitative way whether the presence or absence of biophysical barriers explains the strength of the genetic structure. For instance, the majority of papers found genetic structure and biophysical barriers, which was then classified as the matching category ‘structure–barrier’. In some studies, the data did not allow an assessment of match and were then classified as ‘unclear’. Based on these classifications we could then further investigate which features contribute to a good fit between biophysical modelling and genetics, i.e. species biology, environment and specifications of the genetic assessment or the biophysical model. While 59% of the papers were classified as IBO in assessment 1 (table 1) and 59% classified as ‘structure–barrier’ in assessment 2 (table 2), there is no clear relationship between the two type of assessments (figure 3*a*).

**Table 1.** Description of the classification scheme used for the assessment of fit between biophysical and genetic methods. IBO, isolation-by-oceanography; IBD, isolation-by-distance; AEM, asymmetric eigenvector maps.

classification	description	percentage of papers
*power of combined approach*		
IBO	studies with equally or better fit than IBD (e.g. Mantel test, AEM, networks) to predictions from the biophysical model, were classified as IBO	59
IBD	studies with a significant IBD pattern (e.g. using Mantel test), but with poor correspondence with predictions from the biophysical model	19
unclear	for many studies, e.g. where statistical tests were missing (e.g. assessed by eye, networks or genetic models), it was not possible to make a clear assessment, and those studies were classified as unclear	22

**Table 2.** Description of the matching score system used to classify the type of structure observed with biophysical and genetic methods.

classification	description	percentage of papers
*matching categories*		
weak structure–barrier	studies that detected no genetic structure using metrics of genetic distance or PCAs, while the biophysical model indicated barriers to dispersal, e.g. from network analysis	11
structure–no barrier	studies where genetic structure was identified from significant metrics of genetic distance or by clusters in PCAs, but biophysical modelling did not indicate barriers to dispersal	0
weak structure–no barrier	studies detecting no genetic structure using metrics of genetic distance or PCA, and when biophysical modelling indicated no barriers to dispersal, e.g. from network analysis	14
structure–barrier	studies where genetic structure was identified from significant metrics of genetic distance or by clusters in PCA, and when the biophysical modelling supported the genetic pattern by Mantel tests, networks, AEM or in rare cases by eye	59
unclear	studies that did not report a clear pattern, or a few cases where our assessment of genetic structure and biophysical modelling differed from the conclusions by the authors of reviewed papers	16

### (a) Species biology

**Figure 4.**
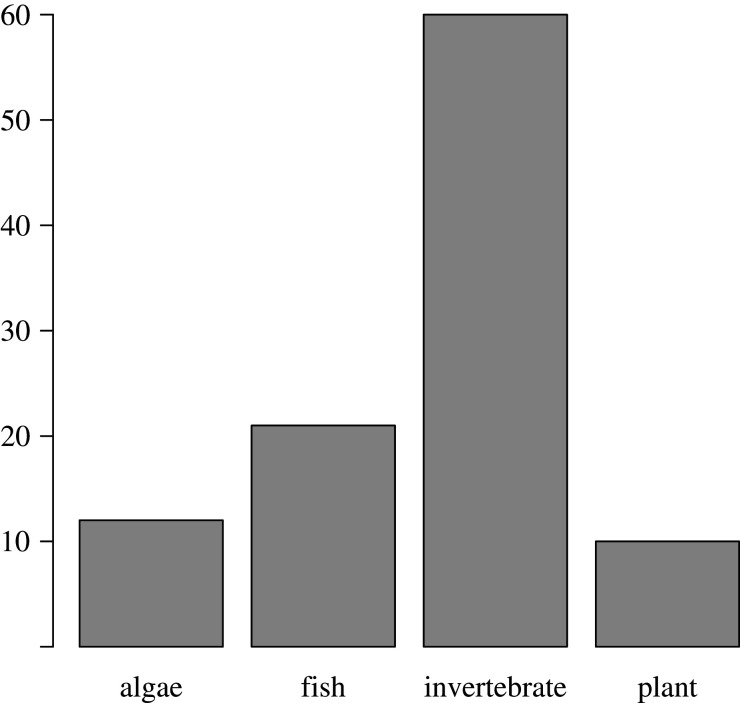
Taxonomic distribution of the assessed species in the 87 surveyed publications.

In terms of species biology, we assessed taxonomy, reproductive strategy and pelagic larval duration (PLD). Altogether the 87 papers assessed 90 different species, of which the majority were invertebrates (figure 4). The taxonomic diversity was high although most studies, obviously, included species where dispersal was assumed to be influenced by the ocean circulation. One functional group largely missing is holoplanktonic species with only few examples (e.g. [22]). The distribution of high-level taxonomic groups is similar among the three categories in assessment 1 (table 1, IBO: 12% algae, 25% fish, 55% invertebrates, 8% plants; IBD: 17% algae, 17% fish, 61% invertebrates and 6% plants; unclear: 13% algae, 12% fish, 64% invertebrates and 16% plants).

The majority of species are reproducing exclusively sexually (63%), but a considerable proportion (35%) of the assessed species are facultatively sexually reproducing, i.e. have sexual and clonal reproduction. This has important implications also for the potential fit to biophysical modelling, as generally only one of the reproduction modes disperses with ocean currents. For instance, in seagrasses, clonal reproduction is via rhizome extension, at the scale of centimetres per year, while fruits or seeds attached to shoots (sexual reproduction) can potentially be dispersed for hundreds of kilometres. The amount of sexual versus clonal reproduction at a specific site may therefore play a role for the geographical scale of dispersal and the fit with biophysical modelling. The situation is very similar for corals and some facultatively sexually reproducing algae, also included in this survey. In fact, the proportion of species that reproduce both sexually and clonally is similar in the IBO and ‘unclear’ categories (33% for IBO and 35% for unclear), but higher for IBD (42%). This could reflect the fact that in facultatively sexually reproducing species, biophysical dispersal modelling does not catch very local dispersal by clonal reproduction, and is therefore less realistic—at least if sexual reproduction rates are low. There were no strong patterns in regards to PLD among the three categories in assessment 1, but all six cases with a maximum tested PLD above 200 days were assessed as ‘unclear’.

With respect to the type of fit between genetic and biophysical methods according to assessment 2 (table 2), species biology seemed to explain parts of the matching pattern, as all plant and all but one seaweed studies received the matching category ‘structure–barrier’. In addition, organisms that are facultative sexually reproducing did not occur in the category ‘weak structure–no barrier’.

### (b) Environmental features

The geomorphology of the seascape varies greatly depending on the land–sea contour and the bathymetry. Increased complexity of geomorphology may reduce water transport and biological dispersal, cause asymmetric gene flow, and promote genetic differentiation [23]. We assessed coarsely if studies were performed in areas with simple or complex geomorphology ranging from straight-line coasts to areas with archipelagos, fjords or complex bathymetry. The majority of studies were carried out in areas that exhibit a complex geomorphology (complex: 65%, simple: 35%), i.e. dispersal was not constrained along a linear coast as would be assumed under IBD models, but exhibited for instance seasonal changes in directionality, local eddies or the presence of many islands, fjords or river outflow.

Strong asymmetry of biophysical dispersal, i.e. a situation where dispersal from A to B was much more likely than from B to A, seems to complicate assessments: the vast majority of ‘unclear’ cases show asymmetry in dispersal probability in biophysical models among the studied sites, with less asymmetry in IBD and IBO.

### (c) Population genetics/genomics

The first studies used gene markers, for instance CO1, but overall, the most common marker used were microsatellites (61%), with a median of 12 loci. Microsatellites also remain the method of choice in many current papers: of the reviewed papers that were published in 2020, 38% used microsatellites. However, the majority of studies (56%) from 2020 used single-nucleotide polymorphisms (SNPs), which only recently initiated seascape genomics. The first study that used biophysical modelling in combination with population genomic assessments using SNPs was published in 2016 [24]. Between 2016 and 2021 the mean number of SNPs used was 6335. Choice of marker type did not seem to influence fit between genetics and biophysical models (60% of papers using microsatellites found IBO, 57% of publications using SNPs found IBO and 56% of papers that used one or few gene markers found IBO; however, unclear cases were highest with single/few genes (39%) compared to 16% in papers using microsatellites and 29% in papers using SNPs). For assessment 2, marker type played a role, as only few papers that used single genes were classified as ‘structure–barrier’ (17%) compared to publications using microsatellites (70%) and SNPs (62%). Single gene markers used in early studies seem to contain insufficient information about recent genetic processes as the fit to predictions from biophysical models was generally poor. This is expected since differentiation in gene markers generally reflects processes in deeper time. Genomics and thousands of SNPs did not provide more power for analyses that look at gene flow based on neutral markers. This is an interesting finding, and elevates the value of past and current microsatellite assessments investigating neutral population structure. This is of course in stark contrast to seascape genomic studies that assess local adaptation, where the increased power coming with more markers, as well as the ability to distinguish putative loci under selection from neutral loci, opens new areas of research. Some papers, unfortunately, did not state clearly how many individuals were assessed in total, but for those that did the median was 431 individuals, with a minimum of 31 individuals and an impressive 3529 individuals for the earliest study included in this survey [9]. The number of individuals sampled did not seem to contribute to a good fit between genetics and biophysics. However, the three publications that assessed more than 2500 individuals all found that IBO explained genetic structure better than IBD alone [9,25,26].

In terms of the number of sampled sites included in each study, the median was 13, with a minimum of 2 and a maximum of 77 in yet another early study [27]. The number of sites included in an assessment does not explain differences in fit of genetics to the biophysical modelling, which is perhaps explained by the high number of sites included in most studies.

In terms of geographical scale, 11 different categories were defined ranging from fine-scale (1–10s of km) to large scale (10 000s of km). Most assessments sampled sites 10–1000s of km apart (32%), followed by 10–100s of km (21%) and 1–100s and 100–1000s of km apart (10%, respectively). The distance among genetic sampling sites seemed to play a role in explaining the fit between genetic and biophysical modelling. Assessments with a good fit showed a higher proportion of lower maximum sampling distance at the scale of 100s of km, rather than 1000s of km. Some caution is therefore called for when the scale of genetic sampling is very large (greater than 1000 km), as such sampling scales often show genetic patterns well explained both by IBD and by modelled dispersal limitations (IBO). More importantly, large-scale genetic differentiation is likely reflecting processes in deeper time [28], and may be more likely a result of ancient refugia or plate tectonic movements, rather than barriers to current/recent dispersal probabilities. One recommendation is to use nested designs if going beyond the ocean meso-scale (100–500 km). Demographic analyses may be further used to test if recent dispersal within the study domain is a likely process that explains contemporary genetic patterns (e.g. [29]).

### (d) Biophysical modelling

Biophysical modelling of dispersal generally consists of two components—an oceanographic circulation model hindcasting ocean current velocities, which are then used to drive a particle-tracking model that simulates the dispersal of biological propagules. All but one study [30] used three-dimensional circulation models to drive the simulations of dispersal. Piggott *et al*. [30] instead used a database of empirical surface flow based on remote sensing analyses. A range of Lagrangian, particle-tracking routines were used to simulate the dispersal of propagules, although this was not always well documented. The most common biological traits in the models included seasonal release, PLD and drift of particles within a specified depth interval, often in surface waters. Only a few studies (7%) combined particle tracking with individual-based modelling of behaviour, mainly diurnal [31] or ontogenetic vertical migration [25], and even larval growth (e.g. [32]). As both flow speed and direction, particularly in coastal currents, often vary with depth, the vertical position of pelagic larvae may critically affect their dispersal [33]. Results from biophysical model simulations indicate that vertical migration generally seems to reduce the overall dispersal distance [33,34]. While vertical migration behaviour is not relevant for all assessed species, considering vertical migration behaviour where relevant can likely improve the fit between genetics and biophysical modelling [16]. Six of the seven papers that included vertical migration behaviour in their dispersal models found that IBO showed the best fit. This strongly argues for more realistic biophysical models that take into account more aspects of the species biology. A major constraint is the lack of information about larval behaviour for most species.

Most studies used a single structured grid (95%) with a few studies employing nested or unstructured grids. The horizontal resolution of the biophysical models ranged from 0.08 to 55 km (median: 6.5 km). Most studies used spatial resolutions of 3–10 km, but complex coasts, e.g. with archipelagos, require higher spatial resolution, often less than 1 km (e.g. [35]), which may restrict the extent of the model domain because of current computational limitations. A few studies used an unstructured grid for the computation of the circulation model (e.g. [10]), which may offer a suitable compromise between extent and resolution. There was no clear relationship between model spatial resolution and the match between genetic and biophysical predictions, but the category ‘structure–no barrier’ had on average lower resolution of the biophysical modelling, indicating that important barriers to dispersal were not resolved. Most biophysical models were evaluated for several years allowing for inter-annual variation in physical forcing, e.g. including micro-climatic cycles, which may improve match to observed population genetic differentiation that integrates gene flow over many generations. At the other end, 13% of the assessments were based on a single year. For some of the studies, this is a strength, as they compare specific dispersal events with contemporary observations from genetic methods [9,16]. The number of years that was included in the model did not contribute to explaining fit according to IBD, IBO and ‘unclear’ in assessment 1 (table 1). However, when only 1 or 2 years of biophysical modelling were included, only 38% of studies were classified as category ‘structure–barrier’, while 68% of studies using at least 5 years of model data were classified as ‘structure–barrier’.

Most studies (61%) correlated genetic patterns and biophysical modelling using estimates of single-generation dispersal events. We did not see that studies employing biophysical estimates of stepping-stone/multi-generation dispersal (39%) improved the fit between population genetic and biophysical predictions. Nevertheless, several publications that compared the fit of single versus multiple generation dispersal with spatial genetic structure found an improved fit of stepping-stone dispersal, particularly at the scale of 100s of km [17,36].

### (e) Approach for comparing genetic and biophysical methods

Our analyses showed that there is no clear method to compare genetic patterns with biophysical predictions, and over 20 different approaches were identified in the reviewed literature. A quarter of the assessments also used two to three methods to assess the fit between genetic and biophysical modelling. We identified that most fits were assessed with Mantel tests (including partial, 28%), followed by ‘by eye’ (24%) and comparisons of network topologies (19%). Other important approaches are regressions, mostly linear regressions (14%), and the comparison of the geographical location of genetic and oceanographic barriers (10%).

There was no clear relationship between the method used for assessments and the fit (figure 3*b*), but several interesting features emerged. None of the ‘unclear’ cases used regressions. All assessments that used Moran's eigenvector maps (MEMs) and asymmetric eigenvector maps (AEMs) followed by redundancy analysis (RDA) [37] were classified as IBO. Perhaps these assessments are more realistic than other methods as they either can take into account asymmetric genetic migration, or account at the same time also for other important abiotic and biotic features, such as salinity and temperature differences and habitat availability. Finally, many of the unclear assessments only used one single method, emphasizing that the use of several methods might be appropriate.

## 3. Discussion

This survey of the seascape genetics/genomics literature evidences that it is a rapidly expanding field that can improve the interpretation of spatial genetic patterns integral in many studies of evolutionary processes, species expansions and invasions, source–sink dynamics, and the management and conservation of the marine environment. As the main message, the present survey shows that many recent studies indeed find that predictions from biophysical models explain aspects of observed genetic patterns. This success of including biophysical modelling in seascape genetics is driven by the recent improvement of three-dimensional circulation models also applied to coastal environments and at scales relevant for biological dispersal. The oceanographic part of biophysical models continues to improve by including more physical processes, adding more detailed forcing data and being simulated with higher spatial and temporal resolution. For example, predictions of cross-shelf dispersal close to the coast is influenced by wave-induced water transport (Stokes drift), and this effect could be implemented into circulation models (e.g. [38]). More detailed forcing data, e.g. high-resolution bathymetry and wind fields, can also significantly improve predictions from biophysical models.

The biological part of biophysical modelling—where an individual-based particle-transport model represents the dispersal of propagules—still lacks many biological traits that may affect dispersal trajectories from release to settlement. Some of the reviewed biophysical models included information about habitat distribution constraining the domain for modelled particle release and settlement. If all or most of the habitat is included in the dispersal model it is possible to explore connectivity across multiple, stepping-stone dispersal events, which may be more representative of potential gene flow. More than one-third of the reviewed papers included projections of stepping-stone connectivity (expected dispersal over multiple generations) and some studies concluded that this significantly improved the fit to spatial genetic metrics [17,39,40]. Unfortunately, the mapping of marine habitats is still in its infancy and lags behind the terrestrial environment impeding seascape genetic analyses, e.g. realistic multi-generation dispersal estimates.

For population genetic/genomic assessments in a spatial seascape context, we identified that method development is necessary to calculate recent gene flow based on genomic datasets (see below). A very promising avenue is the inclusion of demographic models estimating how population genetic patterns develop through time while explicitly considering demographic parameters such as population sizes, migration rates, divergence times and perhaps oceanographic modelling results. Demographic modelling is a very powerful tool to understand (i) historical causes of differentiation and (ii) project the genetic structure of the metapopulation into the future that also includes modelled dispersal, particularly also range evolution within species or metapopulations. Only four of the 87 assessed papers used demographic modelling of the past [13,29,41,42], and only two modelled future persistence and evolution of populations [10,11]. This approach using the tool SLiM [43] is especially appealing in seascape genetics/genomics, as it is possible to construct models including many populations. All publications using demographic modelling are very recent, and we expect that many more future seascape genetic/genomic studies will take up these approaches. In particular, the two studies [10,11] that modelled future persistence or adaptation potential are a major step forward, as these demographic models can include effective population sizes inferred from genetics, genetic information and ecological data as well as dispersal probabilities from biophysical models. The power of combining these different sources of data is that recommendations for adaptive conservation management can be provided, for instance in respect to ‘assisted migration’/’evolutionary rescue’ in corals [11] or re-enforcing restoration in seagrass [10].

A critical step is to assess to what extent a biophysical model of dispersal explains a relevant component of observed genetic patterns. The reviewed papers include a range of comparisons and of statistical methods, and this diversity prevented a formal and unified meta-analysis approach. The most common approach is to test for correlations between pairwise *F*_ST_ or similar pairwise genetic differentiation indices and biophysical connectivity metrics, generally dispersal probability. The most common genetic differentiation indices used are *F*_ST_, *G*_ST_ and Jost's *D*, and while the choice of the genetic index may influence the estimated rate of gene flow, we refer to other excellent reviews on this topic [44–47]. Sometimes estimated gene flow (migration rate), for instance, calculated in BayesAss [48], Migrate [49], divMigrate [50] or using assignment tests [51], is used instead of a pairwise genetic differentiation index, which then allows for correlation tests between asymmetric gene flow and asymmetric biophysical dispersal probabilities (e.g. [17,29,52]. The Mantel test is the dominant statistical method to estimate the correlation between genetic similarity or gene flow with modelled connectivity. The Mantel test also allows for a comparison of fit between genetic differentiation and biophysically modelled connectivity versus IBD. However, the Mantel test has been criticized, mainly because it fails to account for spatial autocorrelation structures (e.g. [53]). We believe that the Mantel test applied to asymmetric migration rates and asymmetric dispersal probabilities is still a strong tool for comparing the effect size of the correlation between genetic and biophysical assessments [5]. However, with one big caveat: assessments of migration rates in shallow time remain still poorly developed in genomic datasets, but BayesAss for SNPs (BA3-SNPs) to model gene flow based on thousands of markers has been recently developed [54]. Nevertheless, we identify the need for genomic methods in addition to divMigrate [50], perhaps based on allele frequency spectra, that can assess migration rates on ‘shallow’ time-scales of 1–100 generations.

An alternative way to the Mantel test that also accounts for non-independence of pairwise comparisons is by using a linear mixed model where each site is included as a random effect [40,55], although issues of spatial autocorrelation may persist. More recent alternatives to the Mantel test include AEMs [56], which may be used to extract eigenvectors describing the spatial field of modelled connectivity at multiple scales. These eigenvectors may be used as independent variables in constrained analyses, e.g. RDA, with sampled allele frequencies as the response variables (e.g. [57]). There is also the possibility to calculate network metrics from the connectivity (adjacency) matrix, estimated from biophysical modelling. Network metrics like centrality or weight of edges [58] for each sample site can then be included together with other environmental variables in an RDA with allele frequencies as response variables. A different approach, employed in many surveyed papers, includes comparisons of genetic discontinuities or breaks to biophysically modelled barriers to connectivity. The statistical genetic method STRUCTURE [59] dominated in the identification of genetic breaks, but other methods that try to account for spatial autocorrelation in the genetic data such as BARRIER [60] and TESS [61] were also used [10,17,62]. The geographical locations of genetic breaks were then compared to dispersal barriers predicted by biophysical modelling. For defining oceanographic barriers, several papers used eigenvector spectral analysis of the connectivity matrix to identify network modularity [63,64] revealing regions separated by poor dispersal [17,65–67].

## 4. Conclusion and future perspectives

This survey of the literature and the analysis on fit between genetic and biophysical methods highlighted several areas where future developments could further increase the power and application of seascape genetic studies that use biophysical models and population genetics.

1. Better and more-widely available habitat mapping will improve the predictions from biophysical models particularly in multi-generation models, as has been shown for sessile priority species such as seagrasses [17] and corals [68].
2. Biophysical models can also improve from a more complete inclusion of hydrodynamic processes, and from better knowledge of larval behaviour (and its interaction with hydrodynamics) [16,33].
3. Method development regarding the inference of recent gene flow from whole-genome sequencing will facilitate the calculation of asymmetric migration rates from genomic datasets.
4. Advanced assignment methods based on whole-genome scans will improve the validation of biophysical modelling of current dispersal.
5. Increased uptake of genetic demographic modelling in seascape genetic/genomic studies, will provide additional assessable time layers (the deep past and/or the future) and will improve the applicability of seascape genetic/genomic studies to conservation management, particularly adaptive management [18].
6. For comparing genetic with biophysical approaches in the seascape context, the field needs to narrow down the methods which are used for comparisons between the two approaches. While the last 15 years were an extremely fruitful period for the development/application of over 20 methods, partly sparked by criticism of the Mantel test, this variability in methods makes different studies less comparable and meta-analyses challenging. Nevertheless, we also support the view of the majority of publications, that using more than one method is beneficial for assessments, for instance using two or more of the most commonly used approaches: Mantel test, network analyses and barrier analyses.

## Data accessibility

All information collected from the literature for this survey is shown in electronic supplementary material, SA1. The data are provided in the electronic supplementary material [69].
